# Validity and reliability of the Arabic version of the population postpartum depression literacy scale (PoDLiS): a web-based survey in Saudi Arabia

**DOI:** 10.1186/s12884-024-06245-0

**Published:** 2024-01-06

**Authors:** Deemah AlAteeq, Ebtesam Almajed, Alya AlZabin, Aisha AlOtaibi, Rawan Bin Salamah, Shahad AlDhafyan, Wijdan A. AlMutiri, Asma AlHarbi, Norah AlQntash, Reem AlTamimi, Raghad AlRasheed, Amel Fayed

**Affiliations:** 1 Internal Medicine Department, College of Medicine, Princess Nourah Bint Abdulrahman University, Riyadh, 16711 Saudi Arabia; 2 Family and Community Medicine Department, College of Medicine, Princess Nourah Bint Abdulrahman University, Riyadh, 16711 Saudi Arabia

**Keywords:** Postpartum, Depression, Literacy, Knowledge, Attitudes, General population, Saudi Arabia

## Abstract

**Background:**

Proper knowledge about postpartum depression (PPD) will help recognize symptoms and encourage women to seek the needed professional help. Until now, there has been a scarcity of research on the literacy level of PPD among the Saudi population and the factors affecting it. This study translated the Postpartum Depression Literacy Scale (PoDLiS) into Arabic and evaluated its psychometric properties. Furthermore, the Saudi population’s mental health literacy for PPD and the factors associated with it were examined as a secondary objective.

**Methods:**

This cross-sectional study involved 2,336 participants selected via convenience sampling from all over Saudi Arabia, all of whom willingly agreed to participate. Data collection was done through an online questionnaire using Google Forms, covering sociodemographic characteristics and the Arabic PoDLiS.

**Results:**

The Arabic version of PoDLiS showed acceptable goodness-of-fit between the observed data and the resulting six-factor solution, RMSEA = 0.049, 90% confidence interval RMSEA (0.010–0.050), pCLOSE = 0.742, CFI = 0.962, TLI = 0.940, χ2(270) = 1576.12, *p*-value = 0.742. The total Cronbach’s alpha (α) of the PoDLiS showed acceptable internal consistency, measuring at 0.742. High literacy was seen in married and young participants with a postgraduate degree and sufficient household income who have known someone with PPD. Significantly lower literacy was seen in male, non-Saudi participants and those residing in the central and northern regions of Saudi Arabia.

**Conclusions:**

The Arabic version of the PoDLiS showed good psychometric properties, and it can be used to assess PPD literacy among perinatal women and to examine the impact of PPD awareness programs. Despite reporting relatively good PPD literacy in the Saudi population, there is a persistent gap in participants’ beliefs about available professional help and the attitudes that facilitate recognizing PPD. Our findings highlight the importance of implementing public education campaigns to improve overall knowledge about PPD and promote prevention, early recognition, and treatment of PPD.

**Supplementary Information:**

The online version contains supplementary material available at 10.1186/s12884-024-06245-0.

## Introduction

Postpartum depression (PPD) is the most prevalent psychiatric condition following parturition [1]. Nevertheless, it is underdiagnosed and, therefore, goes untreated [2]. The fifth edition of the Diagnostic and Statistical Manual of Mental Disorders (DSM-5) classified significant depression with postpartum onset as a major depressive episode that begins during pregnancy or within four weeks following childbirth. Collectively, these episodes are known as peripartum episodes [3]. PPD is characterized by low mood, anhedonia, forgetfulness, irritability, anxiety, sleep disturbances, and poor functioning.

Globally, PPD prevalence is estimated to be between 14 and 25% [4]. A literature review conducted in 2022 revealed that the prevalence of PPD increased to 34% during the COVID-19 pandemic [5]. Moreover, A thorough review of the literature by Halbreich and Karkun in which they identified 143 studies reporting the prevalence in forty countries. According to this review, PPD prevalence varies widely across countries, ranging from 0 to 60%, with factors such as ethnicity, socioeconomic status, cultural patterns, supportive practices, differences in mental health perception, and reporting methods accounting for this broad variation [6]. A recent review study included the studies conducted between 2006 and 2020 in Middle Eastern countries showed that the prevalence of PPD is estimated to be 27% [7]. In Saudi Arabia, a cross-sectional study conducted in Riyadh revealed that among the 174 women who participated, 38.5% reported experiencing PPD [8]. Moreover, based on data collected from 279 Saudi women in 2022 during the postpartum period and on follow up to 2 months after delivery, the study determined that 32.8% of Saudi women had PPD. Lower educational status, unemployment, and previous diagnosis of depression were associated with an increased risk [9]. Interestingly, a recent study conducted in 2023 in Riyadh, Saudi Arabia, aimed to assess the prevalence of PPD and its associated factors that included 187 women who gave birth at King Khalid University Hospital which is a tertiary care hospital. This study reported a prevalence of PPD of 50.3%, which is higher than previously published studies [10]. A 2021 study in Saudi Arabia on community awareness of depression in the Jouf region found that only 26.4% of participants correctly answered 75% of the awareness questions [11]. Variations in PPD prevalence are thought to result from differences in study designs, data collection techniques, assessment tools, and cross-cultural variations [12]. The role of family members in recognizing and preventing PPD is crucial during the perinatal period. Therefore, lack of family support was a significant risk factor for PPD [2].

As a consequence of PPD, maternal confidence is negatively affected as well as the emotional, cognitive, and social development of the child [13]. A detailed literature review showed that children of mothers with PPD were more likely to be underweight in the first year of life and have impaired linear growth after the first year of life [14]. A cross-sectional study from Saudi Arabia revealed that only 6.8% of mothers with PPD seek health care. A study conducted by Mirsalimi et al. showed that poor health literacy is considered a major barrier to professional help-seeking [15].

To date, minimal research has focused on the literacy level and factors affecting the literacy of PPD in Saudi Arabia. Previous research in Saudi Arabia on bipolar disorder and depression has emphasized the importance of mental health literacy (i.e., knowledge about mental disorders aid in their diagnosis, management, and prevention). These studies revealed that the public has suboptimal knowledge, which helped move to the next step of creating interventions to ensure optimal understanding of the population to encourage seeking clinical help [16, 17]. Since proper knowledge about PPD will help in recognizing symptoms and encourage women to seek the needed professional help, it is crucial to measure the awareness and attitudes about PPD among the Saudi population to make the appropriate intervention in the future to help reduce this public health problem. Therefore, the current study translated the Postpartum Depression Literacy Scale (PoDLiS) into Arabic and evaluated its psychometric properties [15]. Additionally, our study measured the Saudi population’s mental health literacy for PPD and examined the factors associated with it as a secondary objective.

## Materials and methods

### Setting and sample

A cross-sectional study explored PPD literacy among the Saudi population through an anonymous online questionnaire using Google Forms and a snowball convenience sampling technique. The survey was sent openly to the general Saudi population, the survey link was shared and circulated by all authors across social media platforms (i.e., WhatsApp, Telegram, Instagram, and Twitter), and the participants were encouraged to share the survey link with their personal and professional contacts and share it on social media. Data collection was from January 2022 to September 2022. The eligibility criteria for participants in this study were living in Saudi Arabia, being at least 18 years old, and being willing to participate; no gender restrictions were applied. Based on a power of 0.95 and the expected prevalence of good PPD literacy of 35%, with ± 5% as the margin of error, the minimal sample size needed was 980, as computed by G-Power software [18]. The total sample size comprised 2,336 participants.

### Ethical considerations

The study was conducted in accordance with the Declaration of Helsinki. Before proceeding, approval was obtained from the Institutional Review Board (IRB) at Princess Nourah bint Abdulrahman University (PNU), Riyadh, Saudi Arabia (IRB log number: 21–0481). Written informed consent was obtained from all participants after a brief explanation of the study’s objectives and the eligibility criteria. They were informed that they had the full right to withdraw from the study without any obligation.

### Instrument and validation

The questionnaire consisted of two sections: (a) Sociodemographic data including age, gender, nationality, current residency, marital status, education level, occupational status, income level, and if they had come across women with PPD. (b) Postpartum Depression Literacy Scale (PoDLiS) [15], consisting of 31 items that were validated through content and face validity; it also has acceptable internal consistency (Cronbach’s alpha = 0.78, which ranged from 0.70 to 0.83 for each factor). The final structure of the scale, as suggested by findings of exploratory factor analysis, was supported and confirmed further with confirmatory factor analysis. A value of UniCo (Unidimensional Congruence) and I-Unico (Item Unidimensional Congruence) larger than 0.95 suggests that data can be treated as essentially unidimensional. A value of ECV (Explained Common Variance) and I-ECV (Item Explained Common Variance) larger than 0.85 suggests that data can be treated as essentially unidimensional. A value of MIREAL (Mean of Item REsidual Absolute Loadings) and I-REAL (Item REsidual Absolute Loadings) lower than 0.300 suggests that data can be treated as essentially unidimensional. The 31 items were distributed across seven subscales as follows: (1) Ability to recognize PPD (6 items). Participants were asked about signs and symptoms of PPD; (2) Knowledge of risk factors and causes and their relation to PPD (5 items); (3) Knowledge and beliefs of self-care activity (5 items). Participants were asked about common approaches that are typically recommended. (4) Knowledge about professional help available (2 items). Participants were questioned about their knowledge of health professionals and their services. (5) Beliefs about professional help available (2 items). (6) Attitudes facilitate recognition of PPD and appropriate help-seeking (6 items). Participants were queried about the attitudes that impact the recognition of PPD and willingness to engage in help-seeking behavior. (7) Awareness of how to seek information related to PPD (5 items). Participants were asked about their confidence in seeking accurate information about mental illnesses. Each item was rated on a 5-point Likert scale ranging from 1 to 5 (i.e., 5 = strongly agree and 1 = strongly disagree). The total raw scores were divided by the number of items to calculate the total score for the PoDLiS, which ranges between 1 and 5.

### Translation procedure

After receiving permission from the original developers of the PoDLiS, four native Arabic speakers translated the scale independently into Arabic. A rigorous review process was employed to identify and resolve any inconsistencies, ambiguities, or potential misinterpretations arising from the four independent translations of the PoDLiS. Discrepancies or disagreements identified during this review were meticulously examined and addressed through collaborative discussions with the translators.

A mental health professional and a non-mental health professional reviewed and approved the draft translation of the PoDLiS. Finally, a qualified professional translator back-translated the Arabic draft into English. We refined the draft after comparing the original questionnaire with the back translation. A panel of five experts, including a psychologist, obstetrician, two head nurses of the obstetrics department, a linguist, 20 personal contacts of the authors, a research professor, and a statistician, were recruited to evaluate item comprehensibility. The participants required approximately 10 min to finish the questionnaire. The subjects thought that each item could be understood without modification. The final 31 items of our Arabic version of the PoDLiS were formed from this step and it was unanimously regarded as fluent and easy to understand.

### Statistical data analysis

Means with standard deviations were used to describe the continuous variables. Histogram plots and the Kolmogorov-Smirnov test were applied to test the statistical normality assumption, and Levene’s test was used to test the homogeneity of the statistical variance. Cronbach’s alpha test was used to assess the scale’s internal consistency. Exploratory and confirmatory factor analyses were used to explore construct validity. Exploratory Factor Analysis (EFA) with the maximum likelihood extraction and the parallel analysis (PA) test were used, and the PoDLiS’ closeness to unidimensionality was assessed with the UniCo (Unidimensional Congruence), and I-UniCo (Item Unidimensional Congruence), as well as the MIREAL (Mean of Item Residual Absolute Loadings) tests to assess whether the 31-indicator long PoDLiS can be used to comprise a total score. Pearson’s correlation test (r) was used to evaluate the correlations between the measured concepts. The multivariate linear regression analysis was applied. The association between the predictor independent variables with their dependent variables were expressed as unstandardized Beta (β) coefficients with their associated 95% confidence intervals. The SPSS statistical analysis program (IBM) was used for all data analyses. The statistical alpha significance level was considered at the 0.050 level.

## Results

### The sociodemographic characteristics

The sociodemographic characteristics of the sample are presented in Table 1. A total of 2,336 participated in the survey, with 94.4% being Saudi citizens. An important proportion of the participants was 21–30 years old (42.7%), female (58.4%), unmarried (63%), had a university education (62.8%) and adequate household income (55.4%), residing in the central region (59.8%). Regarding occupational status, 48.2% were students, 28% were employees, 12.3% were housewives, and 11.5% were unemployed. Only 652 participants reported coming across women with PPD (27.9%).

**Table 1:** Sociodemographic Characteristics of the Participants (*n* = 2,336)

Variables	n (%)
1. Age (years)	
≤ 20	571 (24.4%)
21–30	998 (42.7%)
31–40	345 (14.8%)
≥ 41	422 (18.1%)
2. Gender	
Female	1364 (58.4%)
Male	972 (41.6%)
3. Current Residence	
Central region	1398 (59.8%)
Northern region	164 (7%)
Western region	313 (13.4%)
Southern region	213 (9.1%)
Eastern region	248 (10.6%)
4. Nationality	
Saudi	2206 (94.4%)
Non-Saudi	130 (5.6%)
5. Marital Status	
Unmarried	1472 (63%)
Married	864 (37%)
6. Education	
Secondary school or below	720 (30.8%)
Bachelor’s degree	1466 (62.8%)
Postgraduate degree	150 (6.4%)
7. Occupational Status	
Student	1125 (48.2%)
Employee	655 (28%)
Unemployed	268 (11.5%)
Housewife	288 (12.3%)
8. Household Income	
Adequate and saving	683 (29.2%)
Adequate	1293 (55.4%)
Not adequate	212 (9.1%)
Not adequate and indebt	148 (6.3%)
9. Had Come Across Women with PPD	
Yes	652 (27.9%)
No	1684 (72.1%)

### Psychometric properties of the postpartum depression literacy scale (PoDLiS)

#### Reliability

Table 2 presents the internal consistency analysis of the scale. The tested items (= 30 Items of the PoDLiS) showed an acceptable internal consistency when tested combined, Cronbach’s alpha = 0.742, which ranged from 0.700 to 0.826 across domains, denoting that participants had read and understood these items equally reliably in general.

**Table 2:** Internal Consistency Analysis of the PoDLis

	Number of items	Cronbach’s alpha
Attitudes that facilitate recognition of postpartum depression and appropriate help-seeking	6	0.826
Knowledge of how to seek information related to postpartum depression	5	0.821
Knowledge and beliefs of self-care activities	7	0.762
Ability to recognize postpartum depression	6	0.745
Knowledge of risk factors and causes	4	0.700
Beliefs about professional help available	2	0.800
PoDLiS	30	0.742

ITEM7 EXCLUDED (“How likely is it that postpartum depression might be caused by a genetic or inherited problem?”)

#### Construct validity

Table 3 presents the 31 indicators of the PoDLiS that were subjected to Exploratory Factor Analysis (EFA) with the Maximum Likelihood extraction and Promax Rotation to assess its factorial structure and validity. Principal component analysis (PCA) showed that the sample was adequate; the Kaiser-Meyer-Olkin (KMO) = 0.857 and Bartlett’s test of sphericity indicated that the correlation matrix between items is appropriate for the factor analysis (*p* < 0.001). Additionally, the determinant index = 0.001 shows no unwanted collinearity between the indicators. However, item number 7 (How likely is it that postpartum depression might be caused by a genetic or inherited problem?) showed low commonality with an initially extracted variance below 0.2; therefore, it was excluded. The yielded factor analysis with the parallel analysis (PA) test and the scree-cassilith plot indicated the presence of six main factors that may be extracted from the factor solution. However, the unidimensionality tests indicated that these six latent components might not necessarily comprise one upper-order factor. Consequently, they were considered separate latent components. The items that measured “attitudes that facilitate recognition of PPD and appropriate help-seeking” merged under the first factor, with substantive loadings to this factor (well > 0.30 each). The items that measured “knowledge of how to seek information related to postpartum depression” also loaded significantly and saliently to the second factor. The items measured “knowledge and beliefs of self-care activities” also loaded saliently to the third factor. Furthermore, the items that measured the “ability to recognize postpartum depression” coalesced under the fourth extracted factor. Likewise, the items measuring “knowledge of risk factors and causes” loaded significantly and highly to the fifth factor. The remaining two indicators loaded significantly and saliently to the last sixth factor; these indicators measured “beliefs about professional help available”. Participants who scored higher on those six factors tended to measure significantly higher misconceptions about PPD. Additionally, they tended to measure more self-rated awareness of each PPD treatment source, risk factors, and self-care. However, those who scored higher on the perceived misconceptions about PPD therapeutics tended to perceive the treatment as more harmful and vice versa. Generally, individuals who scored lower on those factors perceived each significantly less.

**Table 3:** Exploratory Factor Analysis with Maximum Likelihood Extraction and Promax Rotation of the Postpartum Depression Literacy Scale (PoDLiS)

	Extracted Factors					
	Attitudes that facilitate recognition of postpartum depression and appropriate help-seeking	Knowledge of how to seek information related to postpartum depression	Knowledge and beliefs of self-care activities	Ability to recognize postpartum depression	Knowledge of risk factors and causes	Beliefs about professional help available
24. It is best to avoid women with postpartum depression so that you don’t develop this problem.	− 0.737					
22. Although there are clinics for women with postpartum depression, I would not have much faith in them.	0.713					
25. If I was a woman with postpartum depression, I wouldn’t tell anyone.	− 0.689					
26. I am afraid of what my family and/or friends might think of me for attending psychology and/ or psychiatry appointments.	− 0.684					
21. I think living with postpartum depression is better than going through the ordeal of getting psychiatric treatment.	0.669					
23. Most women who have postpartum depression are violent.	0.507					
30. I can appraise the accuracy of information about postpartum depression on the Internet.		0.810				
29. I can appraise the accuracy of information about postpartum depression on the radio and television.		0.784				
31. I can appraise the accuracy of advice about postpartum depression given to me by friends and family members.		0.697				
28. I know how to use various sources to seek information.		0.596				
27. I know where to seek information about postpartum depression.		0.581				
15. Having a balanced diet is helpful for the prevention or management of postpartum depression.			0.831			
16. Good sleep is helpful for the prevention or management of postpartum depression.			0.816			
14. Religious practices, prayer, and going to holy shrines are helpful for the prevention or management of postpartum depression.			0.596			
12. Physical activity is effective for the prevention or management of postpartum depression.			0.476			
13. Seeking help with tasks like infant care and household chores from intimate partners and family members is helpful for the prevention or management of postpartum depression.			0.388			
18. Psychotherapy (for example, talking therapy or counseling) can be effective in treating postpartum depression.			0.362			
17. Treatment for postpartum depression, provided by a mental health professional, can be effective.			0.261			
2. Sleeping too much or too little may be a sign of postpartum depression.				0.814		
3. Eating too much or losing interest in food may be a sign of postpartum depression.				0.797		
4. Loss of interest or pleasure in activities may be a symptom of postpartum depression.				0.626		
1. Feeling unusually sad and teary may be a symptom of postpartum depression.				0.532		
5. Postpartum depression affects a person’s memory and concentration.				0.346		
6. Symptoms and signs of postpartum depression last for a period of at least two weeks.				0.275		
8. How likely is it that postpartum depression might be caused by stressful circumstances in life (such as the death of a loved one or divorce)?					0.694	
9. How likely is it that postpartum depression might be caused by a lack of social support such as intimate partner support?					0.642	
10. How likely is it that postpartum depression might be caused by a previous history of depression?					0.502	
11. How likely is it that postpartum depression might be caused by a hormonal imbalance?					0.339	
19. Antidepressants are addictive.						0.800
20. Antidepressants cause brain damage.						0.781

Extraction Method: Maximum Likelihood. Rotation Method: Promax with Kaiser Normalization

Note: Item#7 (How likely is it that postpartum depression might be caused by a genetic or inherited problem?) had very low initial extracted communality (< 0.15) and had cross-loaded non-meaningfully to two distinct factors with high residual loading; for this reason, it was excluded from the exploratory factor analysis

Table 4 displays the descriptive analysis with the mean, standard deviation, and rank order of the means of the PoDLiS items. Participants’ top measured PPD literacy subscale was their knowledge and beliefs of self-care activities (M ± SD: 4.22 ± 0.55). The second top score was their knowledge of risk factors and causes (M ± SD: 4.12 ± 0.64). The third-ranked score was their ability to recognize PPD (M ± SD: 3.88 ± 0.63). The fourth-ranked score measured was their knowledge of how to seek information related to PPD (M ± SD: 3.55 ± 0.78). Attitudes that facilitate recognition of PPD and appropriate help-seeking are measured on the fifth-ranked scale (M ± SD: 2.95 ± 0.41). Beliefs about professional help available were the lowest measured aspect (M ± SD: 2.93 ± 1.04).

**Table 4:** Descriptive analysis with the mean, standard deviation, and rank order of the means of the postpartum depression literacy scale (PoDLiS)

	Mean	SD	Rank
**Attitudes that facilitate recognition of postpartum depression and appropriate help-seeking**	**2.95**	**0.41**	
24. It is best to avoid women with postpartum depression so that you don’t develop this problem	1.92	1.14	6
22. Although there are clinics for women with postpartum depression, I would not have much faith in them	3.72	1.21	2
25. If I was in the place of a woman with postpartum depression, I wouldn’t tell anyone	2.44	1.2	5
26. I am afraid of what my family and/or friends might think of me for attending psychology and/ or psychiatry appointments	2.47	1.29	4
21. I think living with postpartum depression is better than going through the ordeal of getting psychiatric treatment	3.78	1.28	1
23. Most women who have postpartum depression are violent	3.39	1.11	3
**Knowledge of how to seek information related to postpartum depression**	**3.55**	**0.78**	
30. I can appraise the accuracy of information about postpartum depression on the Internet	3.46	1.02	4
29. I can appraise the accuracy of information about postpartum depression on the radio and television	3.32	1.04	5
31. I can appraise the accuracy of advice about postpartum depression given to me by friends and family members	3.62	0.99	2
28. I know how to use various sources to seek information	3.83	0.99	1
27. I know where to seek information about postpartum depression	3.53	1.1	3
**Knowledge and beliefs of self-care activities**	**4.22**	**0.55**	
15. Having a balanced diet is helpful for the prevention or management of postpartum depression	4.16	0.86	5
16. Good sleep is helpful for the prevention or management of postpartum depression	4.27	0.81	3
14. Religious practices, prayer, and going to holy shrines are helpful for the prevention or management of postpartum depression	4.28	0.96	2
12. Physical activity is effective for the prevention or management of postpartum depression	4.16	0.83	6
13. Seeking help with tasks like infant care and household chores from intimate partners and family members is helpful for the prevention or management of postpartum depression	4.36	0.82	1
18. Psychotherapy (for example, talking therapy or counseling) can be effective in treating postpartum depression	4.18	0.81	4
17. Treatment for postpartum depression, provided by a mental health professional, can be effective	4.11	0.88	7
**Ability to recognize postpartum depression**	**3.88**	**0.63**	
2. Sleeping too much or too little may be a sign of postpartum depression	3.84	0.97	4
3. Eating too much or losing interest in food may be a sign of postpartum depression	3.96	0.91	3
4. Loss of interest or pleasure in activities may be a symptom of postpartum depression	4.06	0.92	2
1. Feeling unusually sad and teary may be a symptom of postpartum depression	4.15	0.84	1
5. Postpartum depression affects a person’s memory and concentration	3.72	1.01	5
6. Symptoms and signs of postpartum depression last for a period of at least two weeks	3.56	1.02	6
**Knowledge of risk factors and causes**	**4.12**	**0.64**	
8. How likely is it that postpartum depression might be caused by stressful circumstances in life (such as the death of a loved one or divorce)?	4.09	0.95	3
9. How likely is it that postpartum depression might be caused by a lack of social support such as intimate partner support?	4.26	0.89	1
10. How likely is it that postpartum depression might be caused by a previous history of depression?	4.01	0.92	4
11. How likely is it that postpartum depression might be caused by a hormonal imbalance?	4.12	0.86	2
**Beliefs about professional help available**	**2.93**	**1.04**	
19. Antidepressants are addictive	2.71	1.16	2
20. Antidepressants cause brain damage	3.14	1.11	1

Item #7: How likely is it that postpartum depression might be caused by a genetic or inherited problem? Mean ± SD = 3.33 ± 1.10

The Closeness to Unidimensionality tests all agreed that the PoDLiS 30 indicator-long questionnaire could not be considered as essentially a unidimensional scale (UniCo = 0.728, ECV = 0.571, MIREAL = 0.278). These indicators suggest that the PoDLiS subscales may correlate significantly with each other but may not necessarily comprise a higher-order concept that can be computed via a total score from the thirty-one indicators.

The above factor solution also showed great goodness-of-fit between the observed data and the resulting six-factor solution, RMSEA = 0.049, 90% confidence interval RMSEA (0.010–0.050), pCLOSE = 0.742, CFI = 0.962, TLI = 0.940, χ2(270) = 1576.12, *p*-value = 0.742. Therefore, the factor solution with distinct subscale scores was accepted, and these subscale scores were analyzed with multivariate linear regression to assess the significant variables.

### Factors correlated with Postpartum Depression literacy

Table 5 displays the multivariate linear regression analysis of the PoDLiS subscales. Many subscales correlated with each other and with the sociodemographic variables.

**Table 5:** Multivariate linear regression analysis of the postpartum depression literacy scale (PoDLiS) subscales

	Unstandardized β Coefficients	95% CI for β Coefficient		*p*-value
		Lower Bound	Upper Bound	
Attitudes that facilitate recognition of postpartum depression and appropriate help-seeking *				
(Constant)	2.808	2.643	2.973	< 0.001
1. Age (in years)	.001	− .002	.002	.755
2. Sex (Male)	.037	.003	.072	.035
3. Nationality (Non-Saudi)	− .049	− .117	.018	.152
4. Marital state (Married)	− .055	− .107	− .002	.041
5. Occupation (Housewife)	− .055	− .109	− .001	.044
6. Knowledge of how to seek information related to postpartum depression	− .013	− .034	.007	.206
7. Knowledge and beliefs of self-care activities	− .023	− .055	.009	.154
8. Ability to recognize postpartum depression	.032	.004	.060	.027
9. Knowledge of risk factors and causes	− .006	− .035	.023	.685
10. Beliefs about professional help available	.066	.051	.081	< 0.001
Knowledge of how to seek information related to postpartum depression **				
(Constant)	1.853	1.493	2.213	< 0.001
1. Age (in years)	.001	− .002	.003	.682
2. Sex (Male)	− .046	− .111	.019	.163
3. Educational Level	.028	− .025	.081	.308
4. Had Come Across Women with PPD (Yes)	.142	.074	.209	< 0.001
5. Residence (Central region)	− .076	− .138	− .014	.016
6. Knowledge and beliefs of self-care activities	.246	.186	.306	< 0.001
7. Ability to recognize postpartum depression	.214	.160	.267	< 0.001
8. Knowledge of risk factors and causes	.005	− .050	.060	.856
9. Beliefs about professional help available	− .020	− .049	.009	.167
10. Attitudes that facilitate recognition of postpartum depression and appropriate help-seeking	− .042	− .115	.031	.262
Knowledge and beliefs of self-care activities ***				
(Constant)	2.214	1.986	2.443	< 0.001
1. Age (in years)	.001	− .001	.004	.256
2. Sex (Male)	.039	− .003	.082	.069
3. Nationality (Non-Saudi)	− .127	− .209	− .044	.003
4. Marital state (Married)	.069	.007	.131	.029
5. Educational Level (Higher education)	− .044	− .079	− .010	.012
6. Household Monthly Family Income Sufficiency	.027	.003	.051	.030
7. Had Come Across Women with PPD (Yes)	− .038	− .082	.006	.092
8. Ability to recognize postpartum depression	.126	.091	.161	< 0.001
9. Knowledge of risk factors and causes	.311	.277	.345	< 0.001
10. Beliefs about professional help available	− .037	− .056	− .019	< 0.001
11. Attitudes that facilitate recognition of postpartum depression and appropriate help-seeking	− .018	− .065	.029	.457
12. Knowledge of how to seek information related to postpartum depression	.087	.062	.112	< 0.001
Ability to recognize postpartum depression ****				
(Constant)	1.141	.877	1.404	< 0.001
1. Age (years)	− .002	− .005	.001	.135
2. Sex (Male)	− .097	− .144	− .049	< 0.001
3. Marital state (Married)	− .061	− .133	.012	.101
4. Educational Level (Higher education)	.087	.048	.125	< 0.001
5. Occupational status	− .012	− .032	.007	.217
6. Had Come Across Women with PPD (Yes)	.155	.107	.204	< 0.001
7. Knowledge of risk factors and causes	.319	.281	.357	< 0.001
8. Beliefs about professional help available	.058	.037	.079	< 0.001
9. Attitudes that facilitate recognition of postpartum depression and appropriate help-seeking	.076	.024	.129	.004
10. Knowledge of how to seek information related to postpartum depression	.096	.068	.123	< 0.001
11. Knowledge and beliefs of self-care activities	.173	.130	.216	< 0.001
Knowledge of risk factors and causes *****				
(Constant)	1.523	1.255	1.790	< 0.001
1. Age (in years)	− .002	− .004	.000	.032
2. Sex (Male)	− .163	− .209	− .116	< 0.001
3. Nationality (Non-Saudi)	.093	− .002	.189	.055
4. Educational Level (Higher education)	.039	− .001	.078	.055
5. Beliefs about professional help available)	− .011	− .033	.011	.318
6. Attitudes that facilitate recognition of postpartum depression and appropriate help-seeking	− .024	− .079	.030	.379
7. Knowledge of how to seek information related to postpartum depression	− .004	− .033	.024	.768
8. Knowledge and beliefs of self-care activities	.369	.327	.411	< 0.001
9. Ability to recognize postpartum depression	.324	.286	.362	< 0.001
Beliefs about professional help available ******				
(Constant)	1.351	.837	1.865	< 0.001
1. Age (in years)	.004	.001	.008	.023
2. Sex (Male)	− .122	− .211	− .033	.007
3. Nationality (Non-Saudi)	− .247	− .424	− .071	.006
4. Educational Level (Higher education)	.074	.001	.147	.048
5. Residence (Northern region)	− .266	− .424	− .108	.001
6. Household Family Income Sufficiency	.124	.073	.175	< 0.001
7. Had Come Across Women with PPD (Yes)	− .106	− .199	− .012	.026
8. Attitudes that facilitate recognition of postpartum depression and appropriate help-seeking	.502	.403	.601	< 0.001
9. Knowledge of how to seek information related to postpartum depression	− .014	− .067	.039	.596
10. Knowledge and beliefs of self-care activities	− .197	− .279	− .114	< 0.001
11. Ability to recognize postpartum depression	.212	.138	.287	< 0.001
12. Knowledge of risk factors and causes	− .042	− .117	.034	.278

*DV = Attitudes that facilitate recognition of postpartum depression and appropriate help-seeking. Model R = 0.228, Adjusted R-squared = 0.05

** DV = Knowledge of how to seek information related to postpartum depression

*** DV = Knowledge and beliefs of self-care activities. Model R = 0.50, Adjusted R-squared = 0.240

**** DV = Ability to recognize postpartum depression. Model R = 0.529, Adjusted R-squared = 0.276

***** DV = Knowledge of risk factors and causes. Model R = 0.550, adjusted R-squared = 0.296

****** DV = Beliefs about professional help available, Model R = 0.302, adjusted R-squared = 0.10

The mean score of the first subscale, “attitudes that facilitate recognition of PPD and appropriate help-seeking” reflects perceived misconceptions about PPD and its treatments and correlated significantly with three factors and two subscales. Males had significantly higher mean scores on this subscale than females (β coefficient = 0.037, *p* = 0.035). In contrast, married and housewives measured significantly lower mean scores (β coefficient = -0.055, *p* = 0.041 and β coefficient = -0.055, *p* = 0.044, respectively). Furthermore, two subscales (“ability to recognize postpartum depression” and “beliefs about professional help available”) correlated positively and significantly with this subscale (β coefficient = 0.032, *p* = 0.027 and β coefficient = 0.066, *p* = 0.001, respectively).

The mean score of the second subscale, “knowledge of how to seek information related to PPD,” correlated significantly with two factors and two subscales. Participants who had come across women with PPD measured significantly higher mean scores compared to participants who had no relation with women diagnosed with PPD (β coefficient = 0.142, *p* < 0.001). Additionally, participants living in the central region of Saudi Arabia measured significantly lower mean scores on this subscale compared to other regions (β coefficient = -0.076, *p* = 0.016). Furthermore, two subscales (“Knowledge and beliefs of self-care activities” and “Ability to recognize postpartum depression”) correlated positively and significantly with this subscale (β coefficient = 0.246, *p* < 0.001, and β coefficient = 0.214, *p* < 0.001, respectively).

**Fig. 1:**
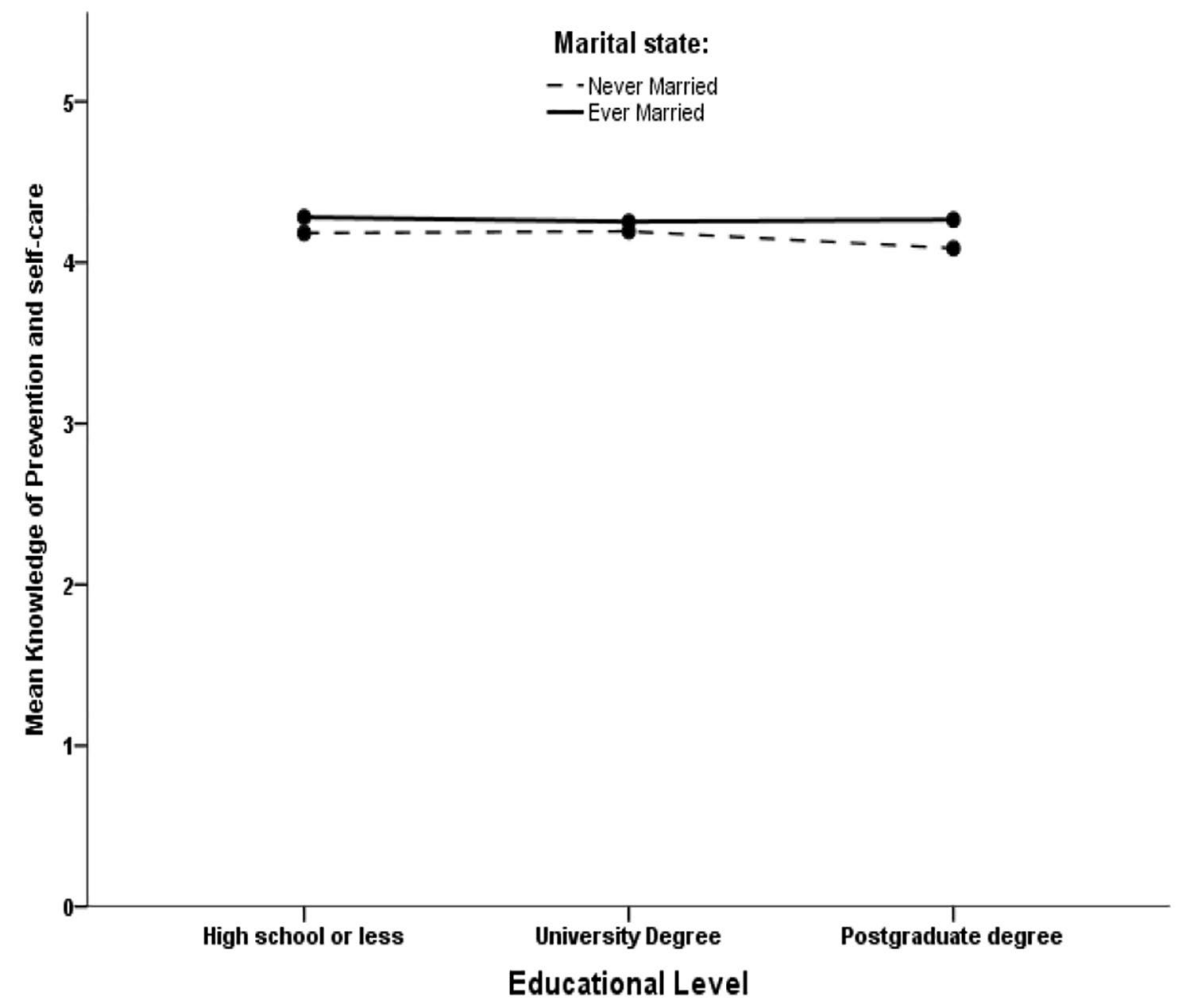
The association between education level and knowledge and beliefs of self-care activities accounting for marital status

The mean score of the third subscale, “Knowledge and beliefs of self-care activities,” correlated significantly with four factors and four subscales. Non-Saudi participants measured significantly lower mean scores than Saudis (β coefficient = -0.127, *p* = 0.003). Married and higher household income participants had significantly higher mean scores on this subscale (β coefficient = 0.069, *p* = 0.029 and β coefficient = 0.027, *p* = 0.030, respectively). Interestingly, highly educated participants measured significantly lower mean scores than those with university or lower education levels (β coefficient = -0.044, *p* = 0.012). However, an interaction test was conducted between marital state and education level in the analysis model, which was non-statistically significant, as seen in Fig. 1. Highly educated participants who were unmarried measured slightly lower mean scores compared to married participants with the same or lower education levels. Furthermore, three subscales (“Ability to recognize PPD,” “Knowledge of risk factors and causes,” and “Knowledge of how to seek information related to postpartum depression”) correlated positively and significantly with this subscale (β coefficient = 0.126, *p* < 0.001, β coefficient = 0.311, *p* < 0.001, and β coefficient = 0.087, *p* < 0.001). In contrast, the “Beliefs about professional help available” subscale correlated negatively and significantly (β coefficient = -0.037, *p* < 0.001).

**Fig. 2:**
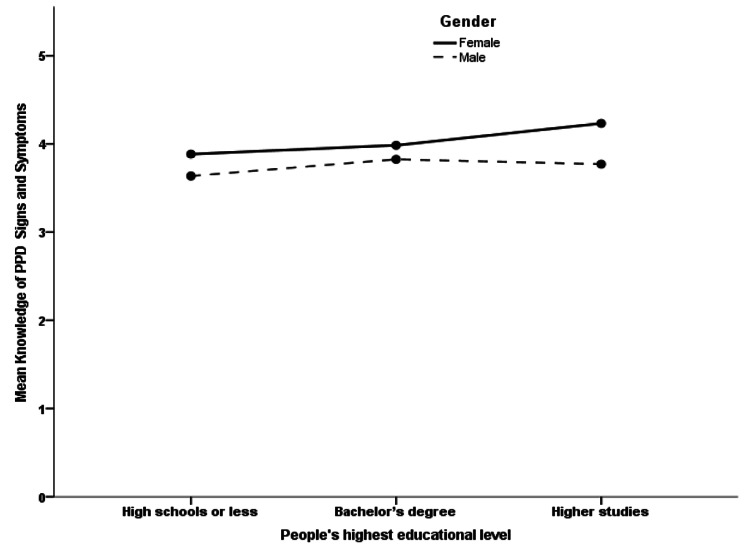
The association between education level and the ability to recognize postpartum depression accounting for gender

The mean score of the fourth subscale, “Ability to recognize PPD,” correlated significantly with three factors and five subscales. Male participants had significantly lower mean than females (β coefficient = -0.097, *p* < 0.001). More highly educated participants measured significantly higher mean scores than university or less educated levels (β coefficient = 0.087, *p* < 0.001). Although the education level tended to rise when the mean score for the ability to recognize PPD tended to rise, females measured significantly higher knowledge than males across all education levels. The interaction effect between gender and education level regarding their ability to recognize PPD score was statistically insignificant, as shown in Fig. 2. Additionally, coming across women with PPD showed a significantly higher mean score than those who had not known a woman with PPD (β coefficient = 0.155, *p* < 0.001). Furthermore, all other five subscales (“knowledge of risk factors and causes,” “beliefs about professional help available,” “attitudes that facilitate recognition of PPD and appropriate help-seeking,” “knowledge of how to seek information related to postpartum depression” and “knowledge and beliefs of self-care activities”) correlated positively and significantly with the mean score this subscale (β coefficient = 0.319, *p* < 0.001, β coefficient = 0.058, *p* < 0.001, β coefficient = 0.076, *p* = 0.004, β coefficient = 0.096, *p* < 0.001 and β coefficient = 0.173, *p* < 0.001, respectively).

The mean score of the sixth subscale, “beliefs about professional help available,” correlated significantly with seven factors and three subscales. Participants’ age, highly educated participants, and participants with higher HHI measured significantly higher mean scores on this subscale (β coefficient = 0.004, *p* = 0.023, β coefficient = 0.074, *p* < 0.048, β coefficient = 0.124, *p* < 0.001). In general, older participants tended to believe more in the additivity and brain damage associated with the use of antidepressants compared to younger people. In contrast, males, non-Saudis, participants residing in the Northern region, and participants who came across women with PPD measured significantly lower mean scores on this subscale (β coefficient = -0.122, *p* = 0.007, β coefficient = -0.247, *p* = 0.006, β coefficient = -0.266, *p* = 0.001, and β coefficient = -0.106, *p* = 0.026, respectively). Furthermore, three subscales (“Attitudes that facilitate recognition of PPD and appropriate help-seeking,” “Knowledge and beliefs of self-care activities” and “Ability to recognize PPD”) correlated significantly with the mean score of this subscale (β coefficient = 0.502, *p* < 0.001, β coefficient = -0.197, *p* < 0.001, and β coefficient = 0.212, *p* < 0.001, respectively). Hence, more significant misconceptions about PPD and its treatment were significantly predictive of higher mean misconceptions about antidepressants. On the other hand, more excellent knowledge and beliefs of self-care activities were significantly predictive of lower misconceptions about antidepressants. A more remarkable ability to recognize symptoms of PPD was significantly predictive of higher mean misconceptions about antidepressants.

## Discussion

To our knowledge, this is the first study that translated the PoDLiS into Arabic and implemented it in exploring the Saudi population’s PPD literacy. After examining the reliability and validity of the scale, it showed acceptable internal consistency and good construct validity with 30 items tapping into six factors evaluating knowledge and attitudes towards PPD and seeking help for mental health. The Cronbach’s α was estimated to be = 0.78, which indicates acceptable internal consistency, indicating that all items contribute to the global construct measured, which is similar to the original version (Cronbach’s α 0.78) Mirsalimi et al. [15], and higher than the Malay version (Cronbach’s α 0.73) [19], but lower than Chinese version (Cronbach’s α 0.862) [20]. This scale is adapted by Mirsalimi et al. [15], comprised of seven factors, and based on Jorm’s original model, which contains six factors [21]. Aligned with a study conducted by Guo P et al. in China, which integrated two dimensions of the original PoDLiS, the preliminary adaptation, referred to as C-PoDLiS, encompassed six domains similar to our study [20]. This is in accordance with Jorm’s conceptualization of mental health literacy. According to the C-PoDLiS, the item-total correlations were found to be significant, indicating a strong relationship to the total scale and relative homogeneity of the items [22]. Furthermore, the study was similar to that conducted by Huang et al., in which six factors were considered in the scale [22]. The PoDLiS scale was developed and validated in the Iranian population, which means that some of the items on the scale may not be relevant to the Saudi Arabian cultural context. Among the six attributes of PPD literacy, this study revealed that the highest mean score was “knowledge and beliefs of self-care activities”, followed by “knowledge of risk factors and causes” and the lowest two were “attitudes that facilitate recognition of PPD and appropriate help-seeking” and “beliefs about professional help available” similar to Huang et al. study where the lowest factor was attitudes that facilitate recognition of PPD [22]. The correlation between these attributes were found to be significant, as greater knowledge and beliefs of self-care activities was significantly predictive of higher knowledge of risk factors and causes and lower misconceptions about antidepressants. While greater misconceptions about PPD and its treatment was significantly predictive of higher mean misconceptions about antidepressants. Furthermore, this study revealed that the other attributes had moderate mean scores including the “ability to recognize PPD and “knowledge of how to seek information related to PPD”. A significant correlation was found between these two factors; greater knowledge about signs and symptoms of PPD was significantly predictive of higher knowledge of how to seek information related to PPD. Similar findings were reported by Mirsalimi et al. [15].

Literacy on the third attribute, “knowledge and beliefs of self-care,” was the top measured component of PPD literacy. This domain includes physical activity, religious practices (i.e., prayer and going to holy places), a balanced diet, getting enough sleep, and seeking help with infant care and household chores from intimate partners and family members; all are thought to be effective in the prevention and management of PPD. It also includes psychotherapy and treatment for PPD provided by mental health professionals. The results of this domain may be related to health professionals’ activities in social media or clinics, awareness campaigns, and advertisements that motivate people to adopt healthy lifestyles. Furthermore, an Australian systematic review reported that that mass media campaign has the power to directly and indirectly influence health-related behaviors [23]. In addition, the mental health promotion efforts done by the Ministry of Health and the National Center for Mental Health Promotion could have contributed to the high level of literacy in this attribute in comparison to other attributes [24, 25]. Also, higher household income was predictive of having more knowledge and beliefs of self-care activities. In contrast, lower income was associated with poor depression literacy [26]. This could be explained by the higher educational expectations and opportunities for people with a higher socioeconomic status [27]. This is in line with prenatal research findings that household income is positively associated with the number of prenatal care visits [28].

Women were significantly more able to recognize the risk factors and causes of PPD (e.g., hormonal changes, lack of social support, financial problems, poor marital relationship, low self-esteem, obstetric characteristics, unsuccessful breastfeeding, and inability to have a vaginal delivery) and the symptoms of PPD (e.g., feeling unusually sad and teary, loss of interest or pleasure in activities, oversleeping or insomnia, memory, and concentration problems). Additionally, women had significantly fewer misconceptions about PPD (e.g., rather live with PPD than go through the ordeal of getting psychiatric treatment, having faith in clinics for women with PPD, the belief that women who have PPD are violent, afraid of what family and friends might think of for attending psychology and/or psychiatry appointments) but more misconceptions about antidepressants. These findings are in line with the existing literature [11, 29–31]. This type of depression specifically targets women, so they may feel more driven to be educated about the symptoms [15]. Moreover, women have higher mental health literacy than men in general. One possible reason for this finding is that men are more likely to suggest self-help treatments for mental illness than professional help and are less likely to seek professional help [32, 33]. Interestingly, a study done in 2023 revealed that the prevalence rate of PPD was 43.4%, and family conflict and lack of support by spouse and family during pregnancy were found to be the strongest predictors of developing PPD [34]. Furthermore, females have higher social empathy rates, which might predispose them to have more awareness [35]. In addition, males have negative attitudes toward help-seeking and higher degrees of personal PPD stigma than females [32, 36]. This gender difference in PPD literacy might be explained by the number of female participants in this study being higher than male participants by approximately 17%. Although women had higher PPD literacy in many attributes, they still had misconceptions about antidepressants, such as being addictive or damaging the brain. This might be due to their anxiety toward the emerging issue of substance abuse in Saudi Arabia [37].

This study found that exposure to women with PPD predicted a significantly greater ability to recognize signs and symptoms of PPD and greater knowledge of seeking PPD information help sources compared to participants who had never known a woman with PPD. Knowledge of how to seek information related to PPD includes where to seek, how to use various sources, the ability to appraise the accuracy of information on the radio, television, and the Internet, and the ability to appraise the accuracy of advice about PPD given to them by friends and family members. However, more than a quarter of the sample reported having personal contact with PPD, which indicates that PPD is prevalent in Saudi Arabia. A systematic literature review conducted in the Arab region found six studies in Egypt, three in each of the UAE, Bahrain, Qatar, and Oman, and two in Sudan, concluded that 1 in every 5 mothers in the Middle East are at risk of developing PPD, which emphasizes the prevalence of this condition [38]. This finding highlights the importance of implementing public education campaigns to improve individuals’ knowledge about PPD to facilitate the early recognition of PPD and enhance the overall quality and access to information on PPD.

Being married was significantly predictive of having greater knowledge and beliefs of self-care activities and had fewer perceived misconceptions about PPD and its treatments. This can be explained by higher chances of having a personal postpartum experience or by being in contact with healthcare providers during pregnancy, which helped in increasing awareness and motivation for self-care activities [39]. Regarding age, younger participants exhibited significantly greater knowledge of PPD risk factors and causes and had fewer perceived misconceptions about antidepressants. A prior study found that older age is associated with more negative beliefs [40]. Similarly, a study investigating age’s influence on anxiety literacy found that the younger age groups showed higher literacy due to having educational programs as their primary source of knowledge [35]. Also, younger age groups receive varying degrees of social encouragement along their paths to mental health care. A study investigating the role of youth mental health websites in promoting young people’s help-seeking behavior indicated that using the websites had helped younger age groups acquire the skills and confidence to seek help if needed [41].

Concerning educational attainment, higher-educated participants predicted a significantly greater ability to recognize PPD. Prior research has demonstrated that better-educated individuals are more likely to have a higher literacy level [26]. On the other hand, high education level was significantly predictive of higher perceived misconceptions about antidepressants. This might reflect a gap in the learning resources and the lack of integration of mental health education courses in universities.

Beliefs about professional help available were the lowest attribute of PPD literacy. This domain includes the belief that antidepressant medications are addictive and can cause brain damage. These findings are consistent with the results revealed by Mirsalimi et al. [15]. Similarly, a Saudi study showed that 46% of the sample believed that antidepressants are addictive [42]. Similar results were reported in studies conducted in Italy, Turkey, India, and Denmark [40, 43–45]. This suggests that the population’s beliefs about antidepressants do not vary much across cultures.

This study showed that Saudi general population had a moderate level of PPD literacy, which is similar to the findings in previous studies [15, 26]. A review by Gabriel & Violato, in which they identified 48 studies, concluded that poor understanding of depression, its causes, and treatment options are the most frequently cited reasons why people delay or do not seek professional help [32]. Analogous to the review, a study conducted in 2023 revealed patients with a history of depression had a 3.6 times higher chance of getting PPD [46]. Similarly, a study on the Saudi population assessing mental health awareness and barriers to seeking professional help revealed that a large proportion of individuals were unaware of the availability of mental health services, a quarter hadn’t sought out any mental health services, and more than half were ashamed of meeting a psychiatrist, indicating the stigma surrounding mental illness in Saudi Arabia [47].

Some potential limitations are present in this study. First, using non-probability sampling could limit our findings’ generalizability. Second, the online distribution of the questionnaire may impact the representatives of the study sample and selection bias is possible in Internet-based studies. However, according to the most recent data from the World Bank Database in 2020, 98% of the Saudi population uses the Internet, which means the Internet is widely available in Saudi Arabia and accessible to nearly everyone [48]. Third, personal and family histories of psychiatric disorders were not assessed in the study, which might have influenced the level of PPD literacy. Finally, as a cross-sectional study, establishing causal relationships between the sociodemographic variables and PPD literacy was not feasible.

We would recommend that more studies be made on PPD to find the points that could improve the lack of knowledge. In addition, implementing public health education campaigns about PPD targeting the general population could help spread knowledge to raise awareness and improve its early recognition. Lastly, doctors and specialists should spread awareness about PPD and the available professional help.

## Conclusion

The Arabic version of the PoDLiS has good psychometric properties, and it can be used to assess PPD literacy among perinatal women and to examine the impact of PPD awareness programs. The findings emphasize the importance of implementing public health education campaigns and educational resources about PPD targeting the general population could help spread knowledge to raise awareness and improve its early detection. Future studies should include interventional research.

### Electronic supplementary material

Below is the link to the electronic supplementary material.

Supplementary Material 1

Supplementary Material 2

## Data Availability

The datasets used and/or analysed during the current study are available from the corresponding author on reasonable request.
