# Supplementary material for: Validity and reliability of the Arabic version of the population postpartum depression literacy scale (PoDLiS): a web-based survey in Saudi Arabia
Source: BMC Pregnancy Childbirth. 2024 Jan 6;24:40. doi: 10.1186/s12884-024-06245-0 (PMC10770988; doi:10.1186/s12884-024-06245-0)
Supplement: Supplementary file 1 — Supplementary Material 1 [file 12884_2024_6245_MOESM1_ESM.pdf]

Postpartum Depression Literacy Scale (PoDLiS)

Arabic version

مقياس معرفة اكتئاب ما بعد الولادة (PoDLiS)

النسخة العربية

عزيزنا المجيب / عزيزتنا المجيبة،

يدرس هذا الاستبيان **معرفتك العامة ومعتقداتك حول اكتئاب ما بعد الولادة.**

لكل سؤال، قم باختيار الإجابة التي تصف معرفتك أو معتقداتك بشكل تام.. الرجاء الإجابة على جميع الأسئلة.

رقم السؤال	السؤال	غير موافق بشدة	غير موافق	محايد	موافق	موافق بشدة
١	قد يكون **الشعور بالحزن وكثرة البكاء على غير العادة** من أعراض اكتئاب ما بعد الولادة					
٢	قد يكون **النوم الكثير أو القليل** علامة من علامات اكتئاب ما بعد الولادة					
٣	قد يكون **تناول الكثير من الطعام أو فقدان الاهتمام بالطعام** علامة من علامات اكتئاب ما بعد الولادة					
٤	قد يكون **فقدان الاهتمام أو الاستمتاع بمعظم النشاطات** من أعراض اكتئاب ما بعد الولادة					
٥	يؤثر اكتئاب ما بعد الولادة على **ذاكرة الشخص وتركيزه**					
٦	تستمر **أعراض وعلامات** اكتئاب ما بعد الولادة **لمدة لا تقل عن أسبوعين**					
	**السؤال**	**غير محتمل اطلاقًا**	**غير محتمل**	**محايد**	**محتمل**	**محتمل جدًا**
٧	إلى أي مدى قد يكون اكتئاب ما بعد الولادة نتيجة **مشكلة جينية أو وراثية؟**					
٨	إلى أي مدى قد يكون اكتئاب ما بعد الولادة نتيجة **ظروف مرهقة في الحياة** (كوفاة محبوب أو طلاق)؟					

٩	إلى أي مدى اكتئاب ما بعد الولادة نتيجة **نقص الدعم الاجتماعي مثل دعم الزوج؟**					
١٠	إلى أي مدى قد يكون اكتئاب ما بعد الولادة نتيجة **اكتئاب سابق؟**					
١١	إلى أي مدى قد يكون اكتئاب ما بعد الولادة نتيجة **اختلال التوازن الهرموني؟**					
	**السؤال**	**غير موافق بشدة**	**غير موافق**	**محايد**	**موافق**	**موافق بشدة**
١٢	**النشاط البدني** فعال في الوقاية من أو علاج اكتئاب ما بعد الولادة					
١٣	**طلب المساعدة من الزوج وأفراد الأسرة في المهام مثل المساعدة رعاية الرضيع والأعمال المنزلية** مفيد للوقاية او العلاج من اكتئاب ما بعد الولادة					
١٤	**الممارسات الدينية** مثل الصلاة مفيدة للوقاية او العلاج من اكتئاب ما بعد الولادة					
١٥	**النظام الغذائي المتزن** مفيد للوقاية او العلاج من اكتئاب ما بعد الولادة					
١٦	**النوم الجيد** مفيد للوقاية او العلاج من اكتئاب ما بعد الولادة					
١٧	**العلاج الذي يقدمه أخصائي الصحة النفسية** لاكتئاب ما بعد الولادة قد يكون فعالًا					
١٨	يمكن أن يكون **العلاج النفسي** (على سبيل المثال، العلاج بالحوار أو الاستشارة) فعالاً في علاج اكتئاب ما بعد الولادة					
١٩	أدوية الاكتئاب تسبب الإدمان					
٢٠	أدوية الاكتئاب تسبب تلف في الدماغ					
٢١	التعايش مع اكتئاب ما بعد الولادة أفضل من المرور بمشقة الذهاب للعيادات النفسية					
٢٢	على الرغم من وجود عيادات لاكتئاب ما بعد الولادة، إلا أنني لا اؤمن بها					

٢٣	معظم النساء المصابات باكتئاب ما بعد الولادة عنيفات					
٢٤	من الأفضل للشخص أن يتجنب النساء المصابات باكتئاب ما بعد الولادة، لكيلا ينتقل الاكتئاب إليه					
٢٥	إذا كنت مكان امرأة تعاني من اكتئاب ما بعد الولادة، فلن أخبر أحدًا بذلك					
٢٦	أخاف مما قد تعتقده عني عائلتي و / أو أصدقائي لذهابي لزيارة أخصائي نفسي أو طبيب نفسي					
٢٧	أعرف من أين أحصل على معلومات حول اكتئاب ما بعد الولادة					
٢٨	أعرف كيفية استخدام **المصادر المختلفة** للحصول على المعلومات					
٢٩	يمكنني تقييم مدى صحة المعلومات على **الراديو والتلفزيون** حول اكتئاب ما بعد الولادة					
٣٠	يمكنني تقييم مدى صحة المعلومات المقدمة على **الإنترنت** حول اكتئاب ما بعد الولادة					
٣١	يمكنني تقييم مدى صحة النصائح التي قدمها لي **الأصدقاء وأفراد الأسرة** حول اكتئاب ما بعد الولادة					

شاكرين ومقدرين مشاركتكم في تعبئة هذا الاستبيان، ممتنين لمنحنا دقائق من وقتكم الثمين.
