# Supplementary material for: Validity and reliability of the Arabic version of the population postpartum depression literacy scale (PoDLiS): a web-based survey in Saudi Arabia
Source: BMC Pregnancy Childbirth. 2024 Jan 6;24:40. doi: 10.1186/s12884-024-06245-0 (PMC10770988; doi:10.1186/s12884-024-06245-0)
Supplement: Supplementary file 2 — Supplementary Material 2 [file 12884_2024_6245_MOESM2_ESM.pdf]

Postpartum Depression Literacy Scale (PoDLiS)

English version

Dear respondent,

This questionnaire deals with your perceived knowledge, beliefs, and skills on postpartum depression literacy. For each question, choose the answer that best describes your knowledge, beliefs, or skills. *Please answer all questions.*

Item number	Items	Strongly disagree	Disagree	Neither agree nor disagree	Agree	Strongly agree
1	**Feeling unusually sad and teary** may be a symptom of postpartum depression					
2	**Sleeping too much or too little** may be a sign of postpartum depression					
3	**Eating too much or losing interest in food** may be a sign of postpartum depression					
4	**Loss of interest or pleasure in activities** may be a symptom of postpartum depression					
5	Postpartum depression **affects a person's memory and concentration**					
6	**Symptoms and signs** of postpartum depression **last for a period of at least two weeks**					
	Items	Not likely at all	Not very likely	Neutral	Somewhat likely	Very likely
7	How likely is it that postpartum depression might be caused by a **genetic or inherited problem**?					
8	How likely is it that postpartum depression might be caused by **stressful circumstances in the life** (such as the death of a loved one or divorce)?					
9	How likely is it that postpartum depression might be caused by **lack of social support such as intimate partner support**?					

10	How likely is it that postpartum depression might be caused by a **previous history of depression**?					
11	How likely is it that postpartum depression might be caused by a **hormonal imbalance**?					
	Items	Strongly disagree	Disagree	Neither agree or disagree	Agree	Strongly agree
12	**Physical activity** is effective for the prevention or management of postpartum depression					
13	**Seeking help with tasks like infant care and household chores from intimate partners and family members** is helpful for the prevention or management of postpartum depression					
14	**Religious practices, prayer and going to holy shrine** are helpful for the prevention or management of postpartum depression					
15	Having a **balanced diet** is helpful for the prevention or management of postpartum depression					
16	**Good sleep** is helpful for the prevention or management of postpartum depression					
17	**Treatment for postpartum depression, provided by a mental health professional**, can be effective					
18	**Psychotherapy** (for example, talking therapy or counselling) can be effective in treating postpartum depression					
19	Antidepressants are addictive					
20	Antidepressants cause brain damage					
21	I think living with postpartum depression is better than going through the ordeal of getting psychiatric treatment					
22	Although there are clinics for women with postpartum depression, I would not have much faith in them					

23	Most women who have postpartum depression are violent					
24	It is best to avoid women with postpartum depression so that you don't develop this problem					
25	If I was in the place of a women with postpartum depression, I wouldn't tell anyone					
26	I am afraid of what my family and/or friends might think of me for attending psychology and/ or psychiatry appointments					
27	I know where to seek information about postpartum depression					
28	I know how to use **various** sources to seek information					
29	I can appraise the accuracy of **information** about postpartum depression on **the radio and television**					
30	I can appraise the accuracy of **information** about postpartum depression on **the Internet**					
31	I can appraise the accuracy of **advices** about postpartum depression given to me **by friends and family members**					

Thank you for participating in our questionnaire. We appreciate the time given for completing this survey.
